# Author Correction: ENTPD1/CD39 as a predictive marker of treatment response to gemogenovatucel-T as maintenance therapy in newly diagnosed ovarian cancer

**DOI:** 10.1038/s43856-023-00290-0

**Published:** 2023-05-05

**Authors:** Rodney P. Rocconi, Laura Stanbery, Min Tang, Luciana Madeira da Silva, Adam Walter, Bradley J. Monk, Thomas J. Herzog, Robert L. Coleman, Luisa Manning, Gladice Wallraven, Staci Horvath, Ernest Bognar, Neil Senzer, Scott Brun, John Nemunaitis

**Affiliations:** 1 University of Alabama at Birmingham, Department of Obstetrics and Gynecology, Division of Gynecologic Oncology, Mobile, USA; 2 Gradalis, Inc, Carrollton, USA; 3 Stat-Beyond Consulting, Irvine, USA; 4 Mitchell Cancer Institute, Division of Gynecologic Oncology, University of South Alabama, Mobile, USA; 5 Promedica, Toledo, USA; 6 Arizona Oncology, (US Oncology Network), University of Arizona, Creighton University, Phoenix, USA; 7 University of Cincinnati Cancer Center, Cincinnati, USA; 8 US Oncology Research, The Woodlands, USA; 9 Gold Mast Consulting, LLC, Chicago, USA

**Keywords:** Cancer, Biomarkers

Correction to: *Communications Medicine* 10.1038/s43856-022-00163-y, published online 29 August 2022.

An author, Luciana Madeira da Silva (LMdS), had inadvertently been omitted from the author list of this article. This author has now been added and the author list is as follows: ‘Rodney P. Rocconi, Laura Stanbery, Min Tang, Luciana Madeira da Silva, Adam Walter, Bradley J. Monk, Thomas J. Herzog, Robert L. Coleman, Luisa Manning, Gladice Wallraven, Staci Horvath, Ernest Bognar, Neil Senzer, Scott Brun, John Nemunaitis’.

LMdS is affiliated with the Mitchell Cancer Institute, Division of Gynecologic Oncology, University of South Alabama, Mobile, USA.

The Author Contributions section has been updated to include LMdS and is now as follows: ‘RPR was responsible for conceptualization, methodology, investigation, resources, writing – review and editing. LS and MT were responsible for conceptualization, methodology, validation, formal analysis, investigation, resources, writing – original draft, review and editing. LMdS was responsible for methodology, data generation, investigation, writing – editing. AW was responsible for methodology, supervision, and writing – review and editing. BJM, TJH, RLC, NS and SB were responsible for supervision and writing – review and editing. LM, GW, SH and EB contributed to investigation, resources and writing – review and editing. JN was responsible for conceptualization, investigation, formal analysis and writing – original draft, review and editing.’

These changes have been made in the HTML and PDF versions of the article.

