# Supplementary material for: ENTPD1/CD39 as a predictive marker of treatment response to gemogenovatucel-T as maintenance therapy in newly diagnosed ovarian cancer
Source: Commun Med (Lond). 2022 Aug 29;2:106. doi: 10.1038/s43856-022-00163-y (PMC9424215; doi:10.1038/s43856-022-00163-y)
Supplement: Supplementary file 2 — Description of Additional Supplementary Files [file 43856_2022_163_MOESM2_ESM.pdf]

Description of Additional Supplementary Files

File Name: Supplementary Data 1

Description: Supplementary Data 1 contains source data for the main figures (Figures 3-5)
