# Supplementary material for: ENTPD1/CD39 as a predictive marker of treatment response to gemogenovatucel-T as maintenance therapy in newly diagnosed ovarian cancer
Source: Commun Med (Lond). 2022 Aug 29;2:106. doi: 10.1038/s43856-022-00163-y (PMC9424215; doi:10.1038/s43856-022-00163-y)
Supplement: Supplementary file 4 — Reporting Summary [file 43856_2022_163_MOESM4_ESM.pdf]

nature portfolio

Corresponding author(s): Nemunaitis

Last updated by author(s): Jul 22, 2022

Reporting Summary

Nature Portfolio wishes to improve the reproducibility of the work that we publish. This form provides structure for consistency and transparency in reporting. For further information on Nature Portfolio policies, see our Editorial Policies and the Editorial Policy Checklist.

Statistics

For all statistical analyses, confirm that the following items are present in the figure legend, table legend, main text, or Methods section.

n/a	Confirmed	
☐	☒	The exact sample size (n) for each experimental group/condition, given as a discrete number and unit of measurement
☐	☒	A statement on whether measurements were taken from distinct samples or whether the same sample was measured repeatedly
☐	☒	The statistical test(s) used AND whether they are one- or two-sided<br>*Only common tests should be described solely by name; describe more complex techniques in the Methods section.*
☐	☒	A description of all covariates tested
☐	☒	A description of any assumptions or corrections, such as tests of normality and adjustment for multiple comparisons
☐	☒	A full description of the statistical parameters including central tendency (e.g. means) or other basic estimates (e.g. regression coefficient) AND variation (e.g. standard deviation) or associated estimates of uncertainty (e.g. confidence intervals)
☐	☒	For null hypothesis testing, the test statistic (e.g. F, t, r) with confidence intervals, effect sizes, degrees of freedom and P value noted<br>*Give P values as exact values whenever suitable.*
☒	☐	For Bayesian analysis, information on the choice of priors and Markov chain Monte Carlo settings
☒	☐	For hierarchical and complex designs, identification of the appropriate level for tests and full reporting of outcomes
☒	☐	Estimates of effect sizes (e.g. Cohen's d, Pearson's r), indicating how they were calculated

Our web collection on statistics for biologists contains articles on many of the points above.

Software and code

Policy information about availability of computer code

Data collection	N/A
Data analysis	R (open source, R Core Team)

For manuscripts utilizing custom algorithms or software that are central to the research but not yet described in published literature, software must be made available to editors and reviewers. We strongly encourage code deposition in a community repository (e.g. GitHub). See the Nature Portfolio guidelines for submitting code & software for further information.

Data

Policy information about availability of data

All manuscripts must include a data availability statement. This statement should provide the following information, where applicable:

- Accession codes, unique identifiers, or web links for publicly available datasets
- A description of any restrictions on data availability
- For clinical datasets or third party data, please ensure that the statement adheres to our policy

Supplementary Data 1 contains source data for the main figures (Figures 3-5) in this manuscript. Additional data that support the findings of this study, and that do not involve proprietary or similar commercial information, will be made available to appropriate parties following an approved data sharing request sent to Laura Nejedlik (lnejedlik@gradalisinc.com) at Gradalis, Inc. Requests that may involve a conflict of interest or a competitive risk maybe declined by Gradalis, Inc. in its sole discretion.

Field-specific reporting

Please select the one below that is the best fit for your research. If you are not sure, read the appropriate sections before making your selection.

☒ Life sciences ☐ Behavioural & social sciences ☐ Ecological, evolutionary & environmental sciences

For a reference copy of the document with all sections, see nature.com/documents/nr-reporting-summary-flat.pdf

Life sciences study design

All studies must disclose on these points even when the disclosure is negative.

Sample size	All patients enrolled on the VITAL study were evaluated (n=91).
Data exclusions	No data was excluded.
Replication	We utilized a commercially available NanoString panel which is designed to control for inter and intra run variability.
Randomization	The VITAL study was a double blind placebo controlled trial. Patient randomization was previously described in Rocconi et al., Lancet Oncology 2020. Randomization was not applicable for this post hoc analysis.
Blinding	The blinding methods were previously described in Rocconi et al, Lancet Oncology 2020. Blinding was not applicable for this post hoc analysis.

Reporting for specific materials, systems and methods

We require information from authors about some types of materials, experimental systems and methods used in many studies. Here, indicate whether each material, system or method listed is relevant to your study. If you are not sure if a list item applies to your research, read the appropriate section before selecting a response.

Materials & experimental systems

n/a	Involved in the study	
☒	☐	Antibodies
☒	☐	Eukaryotic cell lines
☒	☐	Palaeontology and archaeology
☒	☐	Animals and other organisms
☐	☒	Human research participants
☐	☒	Clinical data
☒	☐	Dual use research of concern

Methods

n/a	Involved in the study	
☒	☐	ChIP-seq
☒	☐	Flow cytometry
☒	☐	MRI-based neuroimaging

Human research participants

Policy information about studies involving human research participants

Population characteristics	The population demographics are presented in Table 1, including neoadjuvant/adjuvant chemotherapy, stage, age, ECOG status, and residual disease. There were no differences between the two groups.
Recruitment	N/A
Ethics oversight	N/A

Note that full information on the approval of the study protocol must also be provided in the manuscript.

Clinical data

Policy information about clinical studies

All manuscripts should comply with the ICMJE guidelines for publication of clinical research and a completed CONSORT checklist must be included with all submissions.

Clinical trial registration	NCT02346747
Study protocol	https://clinicaltrials.gov/ct2/show/NCT02346747
Data collection	This was a post hoc analysis.
Outcomes	This was a post hoc analysis.
