# Supplementary material for: ENTPD1/CD39 as a predictive marker of treatment response to gemogenovatucel-T as maintenance therapy in newly diagnosed ovarian cancer
Source: Commun Med (Lond). 2022 Aug 29;2:106. doi: 10.1038/s43856-022-00163-y (PMC9424215; doi:10.1038/s43856-022-00163-y)
Supplement: Supplementary file 5 — Supplementary Information [file 43856_2022_163_MOESM5_ESM.pdf]

Supplementary Information: ENTPD1 as a predictive marker of treatment response to gemogenovatucel-T as maintenance therapy in newly-diagnosed ovarian cancer

Rodney P. Rocconi[1], Laura Stanbery[2], Min Tang[3], Adam Walter[4], Bradley J. Monk[5], Thomas J. Herzog[6], Robert L. Coleman[7], Luisa Manning[2], Gladice Wallraven[2], Staci Horvath[2], Ernest Bognar[2], Neil Senzer[2], Scott Brun[8], John Nemunaitis[2]

Supplementary Table 1. Significant genes correlated with OS and RFS from univariate analysis.

	Locus	HR OS	HR RFS	Min Value	Max Value	p value OS	p value RFS
ADORA2A	NM_000675.5	0.099	0.211	-4.22	-2.1	0.0027	0.0006
CCL13	NM_005408.2	0.221	0.423	-6.83	-2.5	0.0034	0.0014
CD79B	NM_001039933.1	0.308	0.447	-6.31	-3.01	0.0051	0.0044
ENTPD1	NM_001098175.1	0.243	0.481	-3.99	0.61	0.0008	0.0006
FAM124B	NM_001122779.1	0.134	0.178	-6.24	-4.24	0.0054	0.0006
GZMB	NM_004131.4	0.452	0.561	-6.97	-1.02	0.008	0.0009
ICOS	NM_012092.3	0.487	0.626	-6.41	-0.49	0.0088	0.0085
IL2RA	NM_000417.2	0.321	0.478	-5.61	-0.74	0.0097	0.0062
LAG3	NM_002286.5	0.409	0.56	-4.86	0.61	0.0054	0.0009
MRC1	NM_002438.2	0.424	0.636	-6.03	-0.41	0.001	0.0096
PDCD1	NM_005018.1	0.314	0.442	-7.49	-3.44	0.006	0.0023
PFKFB3	NM_004566.3	0.206	0.414	-2.51	0.38	.0023	0.0033
STAT4	NM_001243835.1	0.297	0.531	-5.51	-1.62	.0022	0.0039
